# An Adjustable Wireless Backhaul Link Selection Algorithm for LEO-UAV-Sensor-Based Internet of Remote Things Network

**DOI:** 10.3390/s24061973

**Published:** 2024-03-20

**Authors:** Rui Chen, Wennai Wang, Wei Wu

**Affiliations:** 1 Key Laboratory of Broadband Wireless Communication and Sensor Network Technology, College of Telecommunication and Information Engineering, Nanjing University of Posts and Telecommunications, Nanjing 210049, China; 2020010203@njupt.edu.cn (R.C.); 20220087@njupt.edu.cn (W.W.); 2 Jiangsu Broadcasting Corporation, Nanjing 210008, China

**Keywords:** Internet of Remote Things (IoRT), unmanned aerial vehicle (UAV), low-earth-orbit (LEO) satellite, backhaul link selection, multiservices support

## Abstract

Internet of Remote Things (IoRT) networks utilize the backhaul links between unmanned aerial vehicles (UAVs) and low-earth-orbit (LEO) satellites to transfer the massive data collected by sensors. However, the backhaul links change rapidly due to the fast movement of both the UAVs and the satellites, which is different from conventional wireless networks. Additionally, due to the various requirements of IoRT multiservices, the system performance should be comprehensively considered. Thus, an adjustable wireless backhaul link selection algorithm for a LEO-UAV-sensor-based IoRT network is proposed. Firstly, an optimization model for backhaul link selection is proposed. This model uses Q, which integrates the remaining service time and capacity as the objective function. Then, based on the snapshot method, the dynamic topology is converted into the static topology and a heuristic optimization algorithm is proposed to solve the backhaul link selection problem. Finally, the proposed algorithm is compared with two traditional algorithms, i.e., maximum service time and maximum capacity algorithms. Numerical simulation results show that the proposed model can achieve better system performance, and the overload of the satellites is more balanced. The algorithm can obtain a trade-off between remaining service time and capacity by dynamically adjusting model parameters. Thus, the adjustable backhaul link selection algorithm can apply to multiservice IoRT scenarios.

## 1. Introduction

The integration of terrestrial space and aerial access networks is a key use of sixth-generation (6G) technology, which is garnering increasing interest from academics and businesses. This integration greatly enhances the capabilities of the Internet of Remote Things (IoRT) [1,2,3]. IoRT networks have gained widespread use, such as remote surveillance systems for monitoring wild animals, natural disasters, and climate change [4,5,6]. Due to the limitation of the geographical environment, it is difficult to deploy terrestrial base stations in mountains, deserts, and other remote areas, but environmental monitoring data are crucial in such areas. Meanwhile, terrestrial base stations cannot change their fixed locations without incurring high cost. To meet the requirements of IoRT, non-terrestrial networks (NTNs) represent a promising future application of wireless networks, especially for extending connectivity to remote and underserved areas and providing new communication options [7]. NTN technologies like satellites, high-altitude communication platforms (HAPs), and unmanned aerial vehicles (UAVs) can bring temporary or long-term connectivity to regions where terrestrial infrastructure is lacking [8,9,10]. In particular, UAVs fly at a low altitude, are equipped with sensors and monitoring equipment, and can be quickly launched to collect the data of IoRT sensors, which are then transmitted to the core network (CN) [11]. However, due to the limitation of the storage capacity and data processing capability of UAVs, the collected data can be conveyed to satellites in space and then transmitted to remote ground stations [12]. Compared with medium-orbit satellites and synchronous satellite systems, LEO satellites have the advantages of low transmission delay, low launch cost, and global coverage. Combining the advantages of the flexible distribution of UAVs and the long-distance transmission of LEO satellites, a network architecture is proposed in which UAVs serve as base stations and low-orbit satellites serve as relays. This structure belongs to fifth-generation new radio (5G-NR) networks and is a kind of integrated access and backhaul (IAB).

However, NTNs have not yet been taken into consideration, and the present standards only specify IABs for the terrestrial domain [13]. UAV-assisted and satellite–terrestrial IAB networks are the two categories of existing research on non-terrestrial IAB networks. A system model is proposed for forward link broadcasts in an in-band IAB HetNet by incorporating UAVs as drone BSs into the IAB network [14]. The work in [15] solved a path selection problem for a multi-hop IAB network using UAV assistance. In order to determine the best path scheduling strategy and maximize the overall transmission rate, the authors devised and solved a binary linear optimization algorithm. A satellite–terrestrial IAB network design for data offloading was examined in [16]. In order to optimize the overall backhaul capacity, the authors suggested modifying the swap-matching method. Above these studies, the aim is to design the optimal network deployment to achieve economic wide-area connectivity. However, due to the mobility of both the UAV BSs and the LEO relays, there are many crucial challenges to the backhaul links.

The fast and continuous movement of both UAVs and satellites results in rapid topological changes, which break data transmission and degrade system reliability [17]. Moreover, the deployment of UAVs varies based on the specific circumstances and needs of IoRT sensors [9,18]. For example, in rescue operations following an earthquake, UAV-assisted remote control varies from a few kilometers to several hundred kilometers around, whereas UAVs are distributed on multiple lines for the inspection of outdoor high-voltage transmission lines. Therefore, backhaul link selection for a UAV to a group of LEO relays is very complicated.

In addition, due to the use of IoRT technology in various applications being diverse, there are different requirements for quality of service (QoS). For example, a large-scale IoT system in a disaster situation needs more channel capacity and a high throughput [19]. Meanwhile, during the remote control of rescue robots and other rescue equipment, it is necessary to provide constant services and video streaming services. Moreover, the multimedia traffic for the UAV-assisted emergency care communication networks requires both high reliability and high throughput [20,21].

Recently, UAV-LEO integrated networks have been suggested in several studies. The work in [11] integrated UAVs with LEO satellites in emergency areas without ground facilities to meet the QoS requirements of different users. The work in [22] analyzed LEO satellite access time, and the LEO satellite orbit direction mainly affects the path planning of the UAV and minimizes the total energy consumption. In [23], the authors employed multiple UAVs as relays, for which deployments and relay schedules were optimized for maximizing the system’s energy efficiency along with the power allocation. The work in [24] proposes an integration of LEO-Sat and UAVs for post-disaster assistance. It solves the problem of efficient UAV distribution with fairness and budget constraints. However, the LEO-Sat bandwidth resources are based on the average traffic demands of the LEO-UAV links. The various demands of multiservice traffic are not considered. The work [25] investigates the computational tasks and resource allocation in a UAV-assisted multi-layer LEO satellite network. A DDPG-LSTM-based task offloading and resource allocation algorithm is proposed to solve the problem. According to the above review, most of the existing works focused on the problem of the UAVs’ distribution, energy allocation, and bandwidth resource allocation. As the size of the constellation increases and the overlapping range of satellites increases, the selection problem of associated satellites has research value [26]. Meanwhile, the selection method of backhaul links between UAVs and LEO satellites has significant influence on the system performance. However, the selection problem of the backhaul links between UAVs and LEO satellites is rarely discussed in existing works.

Moreover, joint quality factors should be designed to satisfy the various demands of multiservice IoRT sensors in the backhaul link selection algorithm. There are several studies about the service quality of UAV-aided or other wireless networks. The work in [27] jointly optimizes energy and throughput through revenue and cost components to boost radio capacity in hotspot zones in UAV-aided cellular networks. The work in [28] proposes a robust optimization approach to solve the capacity uncertainty in communication links and takes into account the loss of transmission capacity due to weather conditions. The proper resource allocation between ground-to-UAV and UAV-to-satellite links is proposed to improve network throughput and reduce latency in [29]. Therefore, it is particularly important to study an adjustable backhaul link selection method between LEO satellites and UAVs based on the different requirements of QoS.

For clarity, the main contributions of this work are summarized as follows.

(1) The selection problem of the backhaul links between moving UAVs and LEO satellites is solved. The dynamic topology is converted into a static topology based on a snapshot method, and the backhaul link selection problem is formulated as a constrained optimization problem.
(2) The optimization problem considering the multiservice of IoRT applications is researched. An adjustable indicator for evaluating system performance, joining both persistence and capacity, is introduced to adapt to different scenarios. Also, it is the optimization objective. A corresponding heuristic algorithm is proposed to solve the optimization problem.
(3) Numerical simulations to validate the effectiveness of the proposed algorithm are conducted. These simulations compare the performance of the proposed algorithm with two conventional methods that focus on maximizing persistence or optimizing received signal strength, respectively. The results demonstrate the superiority of our algorithm, showcasing that the overload of the satellites is more balanced. The algorithm can obtain a trade-off between remaining service time and capacity by dynamically adjusting model parameters.

The rest of this paper is structured as follows: First, the selection problem of the UAV-LEO backhaul links is introduced in Section 2. Then, the derivation, the definition, and the heuristic algorithm of the optimization model are proposed in Section 3. Furthermore covered in Section 4 are evaluations and discussions of the simulation results. Finally, Section 5 contains a summary of the paper.

## 2. The Selection Problem of the UAV-LEO Backhaul Links

### 2.1. Architecture of LEO-UAV-Sensor-Based IoRT Network System

**Figure 1:**
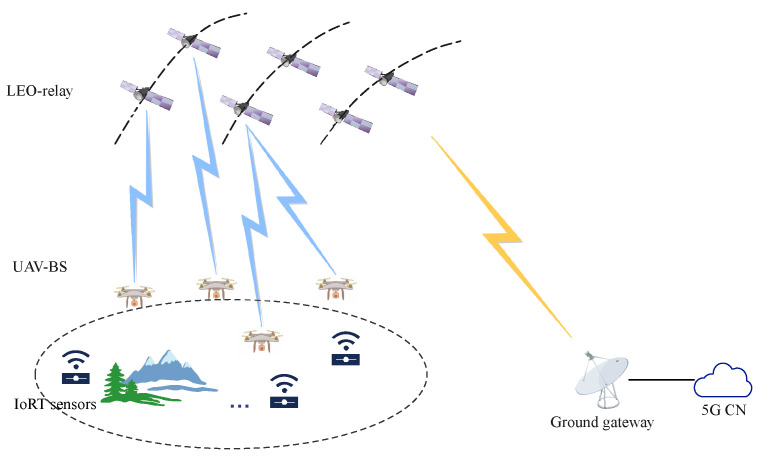
The LEO relay, UAV BS, and IoRT sensor network.

An IoRT network system with wireless UAV-LEO backhaul links is considered, as illustrated in Figure 1, where the 5G core networks are far away from the IoRT sensors. The sensors on the ground transmit the data to UAVs. UAVs are referred to as aerial base stations and work as hubs between the access network and the backhaul network. The part of the UAV–sensor is the access network. Then, the data are transmitted to the core network through the backhaul links. The backhaul links in the IoRT network architecture are from the UAV to the ground gateway. Note that if the ground gateway is remote from the sensors, the data can be transmitted among the inter-satellite links and then downloaded to the gateway. LEO satellites serve as relays of backhaul networks. Thus, the backhaul links are divided into three parts: LEO-UAV, LEO-LEO, and gateway-LEO. Finally, the LEO-UAV part of the backhaul links is the focus of our research. Specially, the LEO satellites have higher store-and-forward capability to process tasks than the UAVs. Therefore, one LEO satellite can be associated with more than one UAV at the same time.

### 2.2. System Model

With the increasing number of LEO satellites, the footprints of the adjacent satellites form more overlapping areas corresponding to the UAVs. Also, some UAVs fly in these overlapping areas. Thus, there are multiple backhaul links that can be selected by the UAV.

In the association model of the UAV-LEO, multiple backhaul links can be established. At time *t*, the association can be modeled as a graph G(t)=(U,S,E(t)), where E(t) is the set of the backhaul links. One vertex set is the UAVs $U=\{U_{1},U_{2},\ldots ,U_{N}\}$, where N is the number of UAVs. The other vertex set is the satellites $S=\{S_{1},S_{2},\ldots ,S_{M}\}$, where M is the number of satellites. There are multiple selective backhaul links for the UAVs to transmit the data to the satellites. These selective links can be described as a candidate set $H\left(t\right)=\left\{H_{1},H_{2},\ldots ,H_{N}\right\}$. The element $H_{i}$ is an array that identifies the visible satellites for the UAV $U_{i}$. Due to the higher processing capability of the satellites, multiple backhaul links can be connected to the same satellite simultaneously. In other words, one UAV has multiple visible satellites that can be selected for communication. These selected nodes are put in a set F(t)={f(1),f(2),…,f(N)}. It means the *i*th UAV is associated with the f(i)th satellite. These nodes associated with UAVs are the edge *E* of graph *G*.

**Figure 2:**
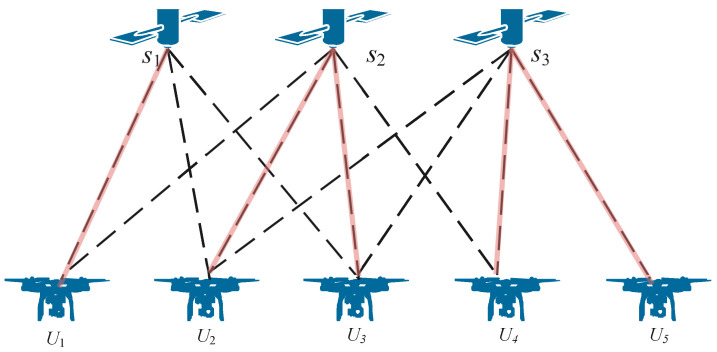
An association method between UAVs and LEO satellites.

We can take an example as in Figure 2 to illustrate the backhaul link model at time *t*. There are three satellites and five UAVs. The dashed lines represent the visibility between satellites and UAVs, and the red line is a kind of selected backhaul link. In the view of the UAVs, the visible satellites can be added to a candidate satellite set H={(1,2),(1,2,3),(1,2,3),(2,3),(3)}. One UAV has multiple visible satellites that can be selected for communication. The end nodes of the links are selected from the candidate set *H*, which are put in a set *F*, such as F={1,2,2,3,3}. Another selected node set is F={2,2,2,2,3}. There are four UAVs connected to the satellite $S_{2}$, and the satellite $S_{1}$ is idle. This situation will cause the system efficiency to decrease. Therefore, different methods of selecting satellites have an important influence on the performance of the system.

## 3. The Optimal Selection Model of the Backhaul Links

In this section, an optimal selection method of the backhaul links between UAVs and satellites is proposed to meet the various requirements of multiservice IoRT systems.

### 3.1. Derivation of the Optimization Model

The introduced system elements include the candidate set H(t) and the selected set F(t). H(t) represents all link connection methods, and F(t) represents one of the selection methods. The latter is a subset of the former. When F(t) is determined, the edges of the graph are also determined. In the graph, the distance of the edge between two vertices can be represented as d(t). Due to the large-scale fading characteristic of electromagnetic waves, the receiving power decreases with the increase in the distance between the satellites and UAVs. Then, according to the Shannon channel capacity formula, the capacity decreases. This is an indicator of system effectiveness. In addition, since satellite motion is regular and predictable, the time during which the satellite stays in the visible range of the UAV can be calculated according to the UAV’s location. This period is called the remaining service time (RST). Once F(t) is determined, the RST is also determined. The greater the remaining service time, the less link switching and the more stable the system is. This is an indicator of system reliability.

In multiservice IoRT scenarios, different scenarios have different needs for both the capacity and RST. Therefore, a comprehensive system performance indicator Q(t) is proposed as a tradeoff between effectiveness and reliability. It is affected by selecting the associated satellite set F(t). Moreover, since the storage and forwarding capabilities of satellites are limited, the number of UAVs connected to each satellite should also be capped. Meanwhile, the selected set F(t) must be in the candidate set. These are two constraints in the optimization model. In summary, the important elements of the optimization model include: the decision variable is the associated satellite set F(t), the objective is to improve the quantity Q(t), and the feasibility constraints are the capped number of satellite connections and the visibility of satellites to UAVs. They are rigorously defined in next subsection.

### 3.2. Definition of the Optimization Model

The objective is to optimize the comprehensive system performance factor Q(t), which contains two adjustable parameters: the RST and the capacity. Both of them are defined as follows:

(1) RST:

Due to the high mobility of the LEO satellites along the orbit, the links between the LEOs and UAVs will be broken. Otherwise, the flexible mobility of the UAVs along different trajectories will also cause the frequent handover of the backhaul links. The more link handover occurrences, the more unstable the system will be. Therefore, RST is an important element for the reliability of the system to indicate handover times. At time *t*, the RST of the nodes $U_{i}$ and $S_{j}$ can be described as $R_{ij}\left(t\right)$, and the total remaining time is the sum of each UAV’s time to be associated with the satellites.

(1) $$R_{sys}\left(t\right)=\sum_{i=1,j=f(i)}^{N}R_{ij}\left(t\right)$$

(2) The capacity of the backhaul links:

In the visible area of a UAV, the selection of backhaul links to transmit the data will influence the capacity of the system. The UAV has better link quality when it is close to the satellite. At time *t*, the information rate of each backhaul link can be described by Shannon’s formula:

(2) $$\begin{array}{cc} C_{ij}\left(t\right)\; & =W\log_{2}\left(1+\frac{P_{r}\left(d_{ij}\left(t\right)\right)}{N_{0}}\right)\\ \; & =W\log_{2}\left(1+\frac{P_{t}G_{t}G_{r}{\left(\frac{\lambda }{4\pi d_{ij}\left(t\right)}\right)}^{2}}{N_{0}}\right) \end{array}$$

The Friis free space formula is used to compute the receiving power, $P_{r}\left(d_{ij}\left(t\right)\right)$, at the satellite end, where $d_{ij}\left(t\right)$ represents the connection distance between the satellite $S_{j}$ and the UAV $U_{i}$. The distance varies with time because of the UAV’s and satellite’s motion. Furthermore, $N_{0}$ is the channel noise, which uses an additive white Gaussian noise (AWGN) model for the interpretation of the channel noise. *W* represents the bandwidth of each UAV, and $P_{t}$ is the transmitting power, which is equal for each UAV. $G_{t}$ and $G_{r}$ represent the transmitting and receiving antenna gains, respectively. The wavelength of an electromagnetic wave is λ. In particular, the square of the distance $d_{ij}\left(t\right)$ determines the inverse proportionality of the receiving power.

The total capacity is the sum of each UAV associated with the backhaul links at time *t*. It can be described as follows:

(3) $$C_{sys}\left(t\right)=\sum_{i=1}^{N}C_{if(i)}\left(t\right)$$

The two parameters refer to the different aspects of the system’s performance. One of the parameters, the RST $R_{sys}\left(t\right)$, reflects the reliability of the system. Meanwhile, the other parameter, the total capacity of the backhaul links $C_{sys}\left(t\right)$, describes the efficiency of the system. To deal with the two attributes on different scales, we can normalize them by min–max normalization.

(4) $$R_{sys}^{\prime }\left(t\right)=\frac{R_{sys}\left(t\right)-\min_{h\in H_{i}}\sum_{i=1}^{N}R_{ih}\left(t\right)}{\max_{h\in H_{i}}\sum_{i=1}^{N}R_{ih}\left(t\right)-\min_{h\in H_{i}}\sum_{i=1}^{N}R_{ih}\left(t\right)}$$

(5) $$C_{sys}^{\prime }\left(t\right)=\frac{C_{sys}\left(t\right)-\min_{h\in H_{i}}\sum_{i=1}^{N}C_{ih}\left(t\right)}{\max_{h\in H_{i}}\sum_{i=1}^{N}C_{ih}\left(t\right)-\min_{h\in H_{i}}\sum_{i=1}^{N}C_{ih}\left(t\right)}$$

where $\min_{h\in H_{i}}\sum_{i=1}^{N}R_{ih}\left(t\right)$ and $\max_{h\in H_{i}}\sum_{i=1}^{N}R_{ih}\left(t\right)$ are the minimum and maximum absolute value of the RST, respectively. Similarly, $\min_{h\in H_{i}}\sum_{i=1}^{N}C_{ih}\left(t\right)$ and $\max_{h\in H_{i}}\sum_{i=1}^{N}C_{ih}\left(t\right)$ are the minimum and maximum absolute value of the total capacity of the backhaul links, respectively. $H_{i}$ is the candidate satellite set corresponding to the *i*th UAV. $R_{sys}^{\prime }\left(t\right)$ and $C_{sys}^{\prime }\left(t\right)$ are the normalized value of the RST and the capacity at time *t*.

The objective is to optimize the comprehensive influence factor Q(t), which contains two adjustable parameters: $R_{sys}^{\prime }\left(t\right)$ and $C_{sys}^{\prime }\left(t\right)$. According to the different requirements, a ratio α,0≤α≤1 is set to adjust the two parameters. Therefore, the comprehensive influence factor Q(t) is:

(6) $$Q\left(t\right)=\alpha R_{sys}^{\prime }\left(t\right)+\left(1-\alpha \right)C_{sys}^{\prime }\left(t\right)$$

Since the selected satellite connected to the UAV has a great influence on the optimization objective, the associated satellite set is the decision variable in the optimization model, as in Equation (7).

(7) F(t)={f(i)},i=1,2,…,N

Typically, one UAV can only choose one backhaul link associated with a satellite in our system. Therefore, only one node can be selected from every array $H_{i}$. The UAV node *U* associated with satellite set F(t) reflects the selection method of the backhaul links. Therefore, the first constraint is set as Equation (9). Moreover, since the storage and forwarding capabilities of the satellites are limited, the number of UAVs connected to each satellite should also be capped. Assume that the number of UAVs connected to each satellite is $P\left(t\right)=\left\{p_{1},p_{2},\ldots ,p_{M}\right\}$, which we can get from *F*. The maximum number of UAVs connected to a satellite can be set as $P_{max}$. The second constraint is set as in Equation (10). Above all, the objective function is to maximize the comprehensive influence factor Q(t) to optimize the performance of the total of the backhaul links from the UAVs to the satellites as follows:

(8) $$\begin{array}{cc} & \begin{array}{c} \max_{F(t)}\;\;Q\left(t\right) \end{array} \end{array}$$

(9) $$\begin{array}{cc} s.t. & f\left(i\right)\left(t\right)\in H_{i}\left(t\right),i=1,2,...,N \end{array}$$

(10) $$\begin{array}{cc} & p\left(j\right)\left(t\right)\leq P_{max},j=1,2,...,M \end{array}$$

The objective function consists of two parts: capacity and service time. The capacity part contains a nonlinear function as formulated by Equation (2). Thus, the optimization model is nonlinear. Because the objective function varies over time, the problem is a dynamic nonlinear optimization problem. In other words, we can break down the optimization problem into simpler sub-problems and solve the sub-problems using a heuristic algorithm. Due to the characteristics of satellite and UAV motion, the snapshot method can be used to make the topology unchanged within a period of time. A heuristic algorithm is used to solve it in the next subsection.

### 3.3. A Heuristic Algorithm for the Optimization Problem

Due to the mobility of both the UAV and the satellite, it is necessary to get the location data and the topological graph during a period of time. Generally, the use of the snapshot method on LEO satellites refers to a specific imaging technique employed for capturing images of the Earth’s surface. Moreover, UAVs can be programmed to follow specific flight paths and to capture images at predetermined locations or on demand. Unlike ordinary aircraft, UAVs have a limited range of motion and can remain still for dozens of seconds. Therefore, UAVs can also use the snapshot method to fix a location or capture images of specific areas on the Earth’s surface. Thus, at each snapshot of a period of time Δt, the objective function can be solved with a general operation.

A heuristic algorithm is presented for adjustable association for UAV-LEO as shown in Algorithm 1.

**Figure 3:**
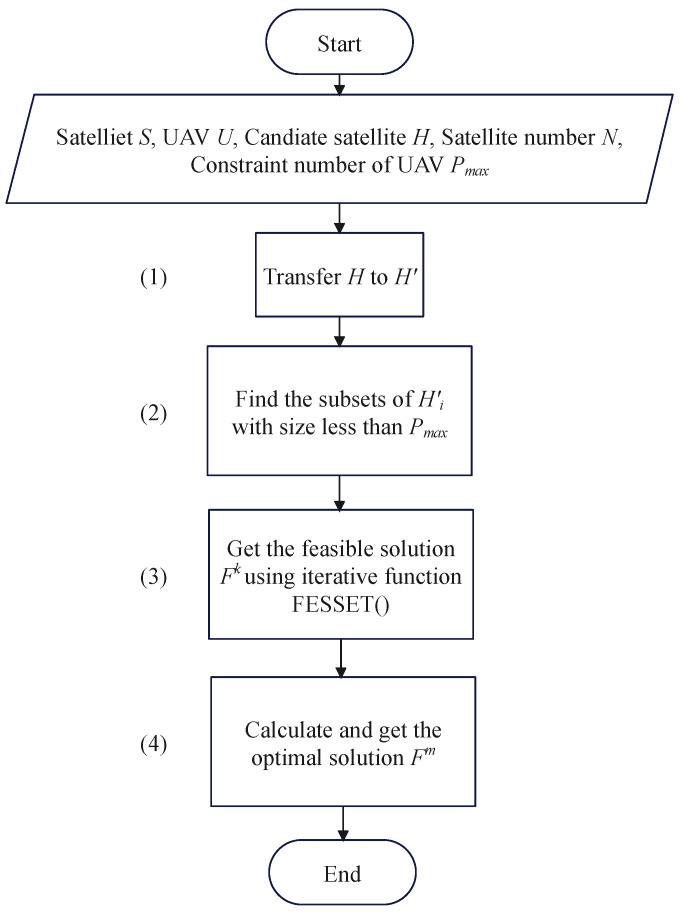
The optimized adjustable satellite association process.

The process is worked as a flowchart in Figure 3. Such a process can be divided into four steps:

Step (1): Transfer the candidate satellite array *H* to the candidate UAV array $H^{\prime }$. Herein, the array $H^{\prime }=H_{1}^{\prime },H_{2}^{\prime },\ldots ,H_{M}^{\prime }$ represents the UAV set that is in the visible area of each satellite $S_{j}$, where $H_{j}^{\prime }$ are the visible UAVs corresponding to $S_{j}$. This process is described in Line 1 in Algorithm 1.

Step (2): Find the subsets of the visible set $H_{j}^{\prime }$: that is, $Su_{j}^{l}$. The subscript of Su means the *j*th satellite, and the superscript denotes the index of the subsets. Specifically, the size of the subset is less than the constraint number of the connected UAVs: that is, $p_{j}\leq P_{max}$. This process is described in Lines 2 to 8 in Algorithm 1 and is used as the constraint in Equation (9).

Step (3): Get all the feasible solutions $F^{k}$. This process is described in Lines 9 to 29. Herein, a feasible set consists of a series of UAVs associated with satellites. We create an iterative function FESSET(·) to search for all the feasible sets in Lines 9 to 22. In the function, a temporary variable fes is set to store the associated UAVs. If fes should satisfy the two constraints in Line 17, the function will be stopped and fes will be added to a feasible solution $F^{k}$. One of the constraints is that fes has to compose the whole UAV set. The other is that a UAV should be associated with only one satellite at the same time. In other words, the elements in the feasible solution should not be duplicated. We use a function Uni(·) to delete the repeated elements in fes. If Uni(fes) is equal to fes, there is no repetition in fes. In this way, we can get many feasible solutions $F^{k}$.

Step (4): Calculate the quality of the system *Q* and get the maximum value. This step is described in Lines 30 to 35 in Algorithm 1.
**Algorithm 1** A Heuristic Algorithm for Adjustable Association.**Input:** UAV set *U*, satellite set *S*, candidate satellite set $H=H_{1},H_{2},\ldots ,H_{N}$, number of UAVs *N*, number of satellites *M*, constraint number of connected UAVs $P_{max}$;**Output:** 
associated satellite set *F*  1:transfer *H* to $H^{\prime }$  2:**for** j=1 to *M* **do**  3:    $b_{j}$ = subsets of $H_{j}^{\prime }$  4:    $p_{j}=$ Size$\left(b_{j}\right)$  5:    **if** $p_{j}\leq P_{max}$ **then**  6:        $Su_{j}^{l}=b_{j}$  7:    **end if**  8:**end for**  9:**function** Fesset(fes,j,k)10:    j=j+111:    **for** each $Su_{j}^{l}$ **do**12:        add $Su_{j}^{l}$ into fes13:        **if** j<M **then**14:           Fesset(fes,j,k)15:        **end if**16:        **if** j=M **then**17:           **if** (fes=U) & (Uni(fes) = fes) **then**18:               **return** fes19:           **end if**20:        **end if**21:    **end for**22:**end function**23:k=124:**for** each $Su_{1}^{l}$ **do**25:    fes=null,j=126:    add $Su_{1}^{l}$ into fes27:    $F^{k}=Fesset\left(fes,j,k\right)$28:    k=k+129:**end for**30:calculate $C_{sys}^{k}$ using 531:calculate $T_{sys}^{k}$ using 332:${\bar{C}}_{sys}^{k}$ = normalize $C_{sys}^{k}$ using 733:${\bar{T}}_{sys}^{k}$ = normalize $T_{sys}^{k}$ using 634:calculate $Q^{k}$ using 835:$F^{m}=argmax\left(Q^{k}\right)$ **return** $F^{m}$

## 4. Evaluation and Discussion

In this section, numerical simulations are first conducted for three different algorithms, i.e., our proposed algorithm, the association method based on the maximum capacity, and the association method based on the maximum service time. Then, their performances are evaluated to validate the effectiveness of our proposed algorithm.

### 4.1. Experimental Settings

The experiments are designed using the configurations tabulated in Table 1. The simulation parameters can be divided into two parts: One is the link budget parameters. The parameters used include the channel bandwidth, effective isotropic radiated power (EIRP), and receiving antenna gain per the SpaceX system’s characteristics [30]. SpaceX’s system uses the Ku-band for communications: specifically, the 12 GHz band used for our simulation system. The other is the satellite and UAV parameters. We follow 3GPP’s example and focus on the LEO constellations at 600 km altitude, for which a satellite moves at a velocity of 7.56 km/s relative to Earth [31]. For the reason that the distance of the UAV from the ground is much smaller than the orbital height, the height of the UAVs is negligible. The deployment of UAVs is different according to different situations. For example, for monitoring highways or high-voltage lines, UAVs are evenly distributed in a line; during an earthquake, UAVs are generally randomly distributed within a radius of several kilometers to tens of kilometers. Thus, we design two experimental scenarios that are Case 1: UAVs are evenly distributed in a line, and Case 2: UAVs are randomly distributed under the footprints of satellites.

Besides the proposed algorithm, two reference algorithms are adopted to compare the performance. One reference algorithm is based on the maximum capacity; the other is based on the maximum service time. These two are commonly used for solving the LEO satellite association problem [32]. For the three different algorithms, we repeated the random association process 1000 times and took the average.

### 4.2. Performance Evaluation

**Figure 4:**
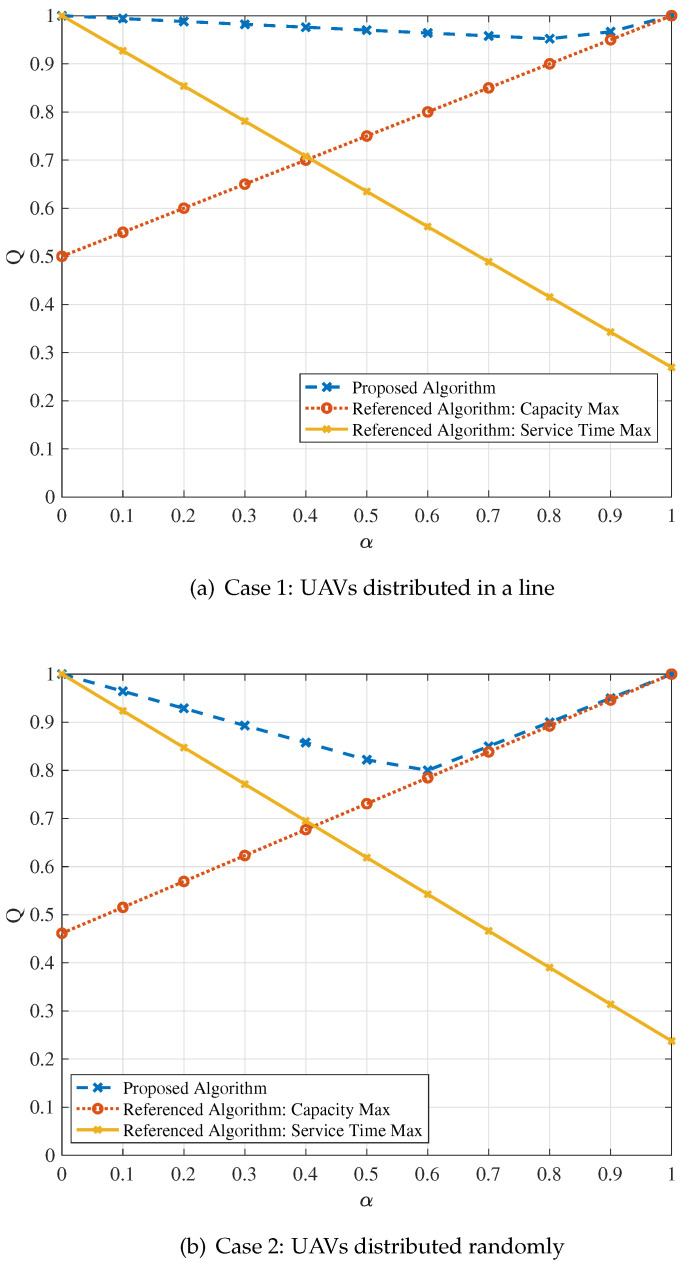
Quality of the system based on the adjustable performance indicator.

The relationships between *Q* and α for two cases are drawn in Figure 4. The first case is for UAVs that are distributed in a line, and the second case is for UAVs that are distributed randomly. In Figure 4, the vertical coordinate *Q* is the adjustable performance indicator. The larger the *Q* value is, the better the exhibited system performance. In addition, the horizontal coordinate α is the adjustable factor. It can be adjusted according to different service requirements. To be specific, for services with higher reliability requirements, the α value should be set higher. For services with higher effectiveness requirements, it should be set lower. For comparison, the curves of the proposed algorithm as well as two referenced ones, including the maximum capacity and maximum service time, are draw in Figure 4. To validate the performance of the proposed model for different services, the adjustable parameter α is set from 0 to 1, and the interval is 0.1.

From glancing at Figure 4a,b, it can be seen that the curve of the proposed algorithm is above the two referenced ones, proving that better overall system performance can be achieved by using the proposed algorithm no matter whether the UAVs are distributed in a line or randomly. For the proposed algorithm, its curve first drops and then rises, but the *Q* value overall remains at a high level, proving that the proposed algorithm can be adapted to different scenarios. For the referenced algorithm based on the maximum capacity, the quality value increases linearly with α. This phenomenon illustrates that the maximum capacity algorithm has better performance for services with high effectiveness requirements, but when the system reliability requirement increases, its performance gradually deteriorates. On the contrary, the quality value decreases linearly for the maximum service time algorithm. It shows that such an algorithm is suitable for services with high reliability requirements.

**Figure 5:**
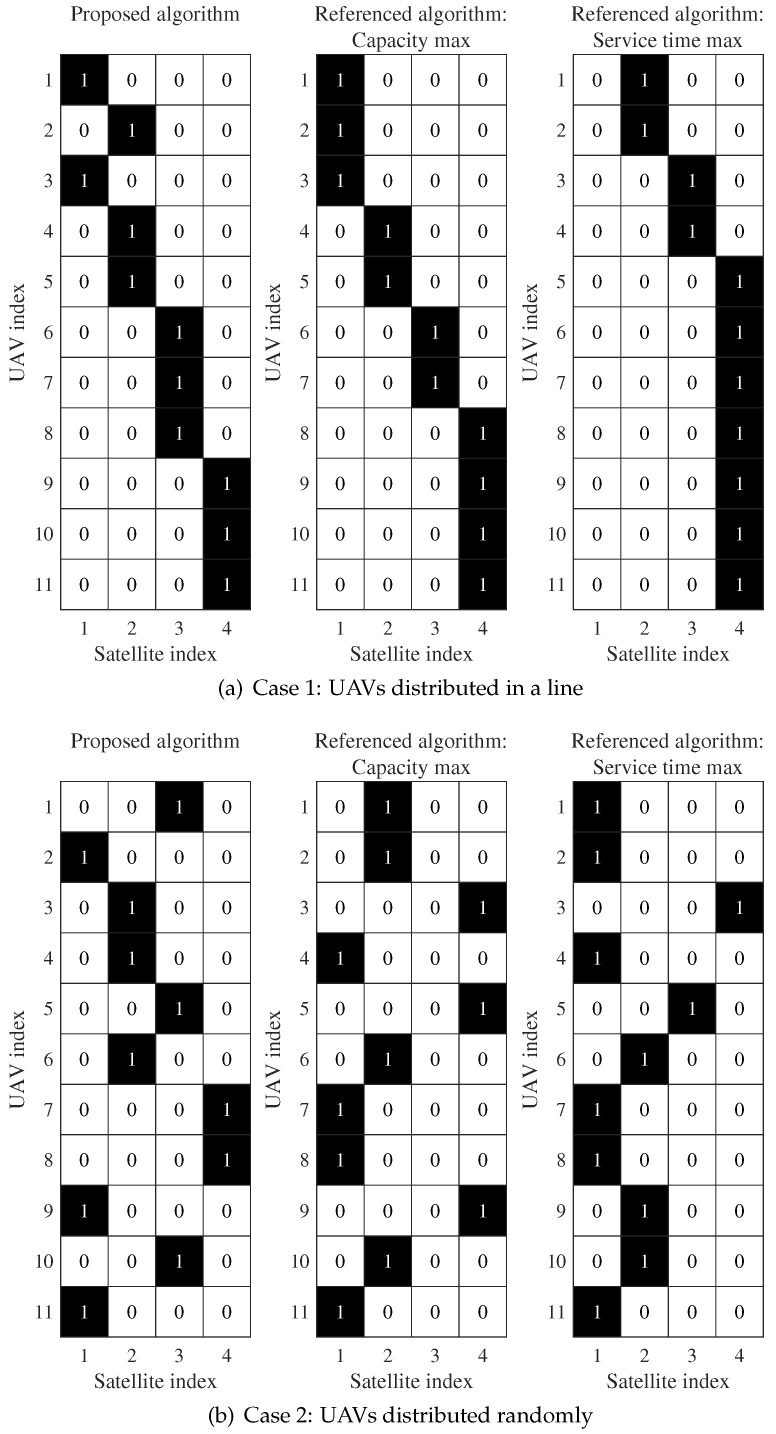
Association pattern of the UAV-LEO.

Then, in order to intuitively display the backhaul link results of different algorithms, as an example, Figure 5 depicts the associated pattern of the UAVs and the satellites for the proposed algorithm and two referenced ones. Herein, the adjustable parameter is set to α=0.5 to simulate services that requires both effectiveness and reliability. Generally speaking, it is better to make the number of UAVs connected to each satellite as even as possible. This has two advantages: One is that the overall load of each satellite is more balanced. Such a method avoids the situation wherein some satellites are overloaded and some satellites are idle. The second is that once a satellite with a heavy load fails, the services provided by the UAVs connected to this satellite will be greatly affected and interrupted. Moreover, the overhead caused during the service switching process will also increase.

In Figure 5a, the number of UAVs associated with the four satellites using the proposed algorithm is 2, 3, 3, and 3. Meanwhile, the number of UAVs is 3, 2, 2, and 4 and 0, 2, 2, and 7 for the referenced algorithms based on the maximum capacity and the maximum service time, respectively. The pattern of the proposed algorithm is more balanced than the two referenced algorithms. For the referenced maximum service time algorithm, the first satellite is idle and the fourth satellite is associated with seven UAVs. In Figure 5b, the number of UAVs associated with the four satellites using the proposed algorithm is 3, 3, 3, and 2, and it is 4, 4, 0, and 3 and 6, 3, 1, and 1 for the two referenced algorithms, respectively. The third satellite is idle when using the maximum capacity algorithm. Meanwhile, the third and fourth satellites are associated with only one UAV when using the maximum service time algorithm. Such an unbalanced pattern will cause the waste of channel resources and the overload of the satellite.

## 5. Conclusions

The challenge of dynamically selecting wireless backhaul links between UAV BSs and LEO relays in IoRT scenarios is resolved. A robust multiple UAV-LEO candidate backhaul link association model that enhances the adaptability and efficiency of the network is established. The optimization problem inherent in IoRT applications, taking into consideration their multiservice nature, and a corresponding heuristic algorithm are proposed. In this problem, an adjustable performance evaluation indicator is adopted that encompasses both the remaining service time and the system capacity. Moreover, comparative analyses against conventional methods are presented. The results show that the proposed algorithm is more balanced and efficient.

Finally, the limits of the present work and directions of research are listed as follows:

(1) The problem of backhaul link selection between UAVs and LEO satellites is considered. However, how to select global links for the satellites, UAVs, and sensors has not been researched. In the future, a three-level model of satellites, UAVs, and sensors could be established for joint optimization to obtain an optimal link selection algorithm.
(2) The free space path loss model is adopted for capacity calculation, and the impact of factors such as weather and season on capacity are ignored. These factors may affect the optimization results. They could be considered by using the corresponding path loss model in our algorithm in future work.
(3) Based on our proposed link selection algorithm, further consideration needs to be given to resource allocation issues such as bandwidth and power. The optimization problem could be modeled and solved according to the various traffic demands of the LEO-UAV links.

## Figures and Tables

**Table 1:** The simulation configuration.

	Item	Configuration
Link budget parameters	Transmitting power ($P_{t}$)	30 dBm
	Bandwidth (*W*)	250 Mbps
	Noise–power spectral density ($n_{0}$)	$10^{-12}$ W/Hz
	Transmitting antenna gain ($G_{t}$)	36.7 dBi
	Receiving antenna gain ($G_{r}$)	32.8 dBi
	Wavelength (λ)	2.5 cm
Constellation configuration	Orbital height	600 km
	Satellite moving speed	7.56 km/s
	Number of UAVs (*N*)	11
	Number of satellites (*M*)	4
	Maximum number of UAVs per satellite ($P_{max}$)	4

## Data Availability

Data are contained within the article.

## Abbreviations

The following abbreviations are used in this manuscript:

UAV	Unmanned aerial vehicle
LEO	Low earth orbit
IoRT	Internet of Remote Things
NTN	Non-terrestrial network
HAP	High-altitude communication platform
CN	Core network
IAB	Integrated access and backhaul
5G-NR	Fifth-generation new radio
QoS	Quality of service
IoT	Internet of Things
RST	Remaining service time
AWGN	Additive white Gaussian noise
EIRP	Effective isotropic radiated power
